# Male-specific Y-linked transgene markers to enhance biologically-based control of the Mexican fruit fly, *Anastrepha ludens *(Diptera: Tephritidae)

**DOI:** 10.1186/1471-2156-15-S2-S4

**Published:** 2014-12-01

**Authors:** J Salvador Meza, Marc F Schetelig, C Silvia Zepeda-Cisneros, Alfred M Handler

**Affiliations:** 1 Programa Moscafrut, SAGARPA-IICA. Camino a los Cacahotales S/N. CP., 30860, Metapa de Domínguez, Chiapas, México; 2 Instituto de Biotecnología y Ecología Aplicada (INBIOTECA). Universidad Veracruzana, Xalapa, Veracruz, México; 3 Center for Medical, Agricultural, and Veterinary Entomology, Agricultural Research Service, U.S. Department of Agriculture, Gainesville, FL, USA; 4 Justus-Liebig-University Giessen, Institute for Phytopathology and Applied Zoology, Giessen, Germany

**Keywords:** Mexfly, gene suppression, Y-linked markers, sexing system, sterile insect technique

## Abstract

**Background:**

Reliable marking systems are critical to the prospective field release of transgenic insect strains. This is to unambiguously distinguish released insects from wild insects in the field that are collected in field traps, and tissue-specific markers, such as those that are sperm-specific, have particular uses such as identifying wild females that have mated with released males. For tephritid fruit flies such as the Mexican fruit fly, *Anastrepha ludens, polyubiquitin*-regulated fluorescent protein body markers allow transgenic fly identification, and fluorescent protein genes regulated by the spermatocyte-specific *β2-tubulin *promoter effectively mark sperm. For sterile male release programs, both marking systems can be made male-specific by linkage to the Y chromosome.

**Results:**

An *A. ludens *wild type strain was genetically transformed with a *piggyBac *vector, pBXL{*PUbnlsEGFP, Asβ2tub-DsRed.T3*}, having the *polyubiquitin*-regulated EGFP body marker, and the *β2-tubulin*-regulated DsRed.T3 sperm-specific marker. Autosomal insertion lines effectively expressed both markers, but a single Y-linked insertion (Y^EGFP ^strain) expressed only *PUbnlsEGFP*. This insertion was remobilized by transposase helper injection, which resulted in three new autosomal insertion lines that expressed both markers. This indicated that the original Y-linked *Asβ2tub-DsRed.T3 *marker was functional, but specifically suppressed on the Y chromosome. The *PUbnlsEGFP *marker remained effective however, and the Y^EGFP ^strain was used to create a sexing strain by translocating the wild type allele of the *black pupae *(*bp*^+^) gene onto the Y, which was then introduced into the *bp*^- ^mutant strain. This allows the mechanical separation of mutant female black pupae from male brown pupae, that can be identified as adults by EGFP fluorescence.

**Conclusions:**

A Y-linked insertion of the pBXL{*PUbnlsEGFP, Asβ2tub-DsRed.T3*} transformation vector in *A. ludens *resulted in male-specific expression of the EGFP fluorescent protein marker, and was integrated into a *black pupae *translocation sexing strain (T(Y^EGFP^/*bp*^+^), allowing the identification of male adults when used in sterile male release programs for population control. A unique observation was that expression of the *Asβ2tub-DsRed.T3 *sperm-specific marker, which was functional in autosomal insertions, was specifically suppressed in the Y-linked insertion. This may relate to the Y chromosomal regulation of male-specific germ-line genes in *Drosophila*.

## Background

A critical component to any prospective field release of a transgenic insect strain is a reliable and robust marking system. Foremost, this is to unambiguously identify the transgenic insects, and to distinguish them from insects in the field, especially in traps that monitor the effectiveness of the release program [1]. For tephritid fruit flies, fluorescent protein markers regulated by the constitutive *polyubiquitin *(PUb) gene promoter are quite effective since the PUb promoter is active in all cell types throughout development (see [2-4]), and for the Caribbean fruit fly, PUb-DsRed.T3 can be visualized unambiguously and detected by PCR in deceased flies maintained in two types of liquid field traps for up to three weeks [5]. A high priority for SIT programs is evaluating the number of wild females that have mated with released sterile males, which can be achieved by sperm-specific markers. Using the spermatocyte-specific *β2-tubulin *promoter [6] to regulate either EGFP or DsRed, fluorescent sperm markers detectable specifically in the female spermathecae, have been developed for several tephritid and mosquito species [7-11].

The Mexican fruit fly, *Anastrepha ludens*, has been successfully transformed using *piggyBac *transposon vectors [12], and specifically by those having fluorescent protein marker genes regulated by the *Drosophila polyubiquitin *and *A. suspensa β2-tubulin *(*Asβ2tub*) promoters [13]. In selecting for dual-marked pBXL{*PUbnlsEGFP, Asβ2tub-DsRed.T3*} transformants, we noted that in autosomal integrations, as determined by segregation analysis, males and females expressed EGFP in the body while only males expressed testis-specific DsRed. However, one line expressed EGFP specifically in males and not in females, suggesting a Y-linked integration, but the expected testis-specific expression of DsRed was not apparent. Here we provide data showing that remobilization of the Y-linked insertion to autosomal sites restores *Asβ2tub-DsRed.T3 *expression, indicating that Y-specific suppression of the *Asβ2-tubulin *promoter may be occurring.

Sex-specific fluorescent protein markers, such as those linked to the Y-chromosome (or Z-chromosome in moths), or whose expression is controlled by a sex-specific promoter or intron-splicing mechanism, can be used for sexing strains previous to release [14,15]. This is particularly advantageous for SIT [16] where sterilization and release of females with males is highly inefficient. However, current sorting systems for fluorescent-marked larvae (or eggs) are not efficient enough for most current fruit fly sterile release programs [9], and automated sexing systems usually rely on pupal color markers (which is combined with an embryonic temperature-sensitive lethal system only in *Ceratitis capitata *[17]). Sex-specificity is achieved in these strains by having the wild type color marker gene translocated to the Y-chromosome, while the homozygous autosomal recessive mutation exists in both males and females [18,19]. Thus, the normal wild type pupal color in males can be distinguished from the mutant color phenotype in females, which has been achieved for the mexfly using the *black pupae *(*bp*) mutation [20,21]. While this system is highly effective for sex separation previous to release, identifying released male adults still depends upon fluorescent powders that are not totally effective, and a health risk for workers [22]. Therefore, male-specific fluorescent protein markers are still the most effective and safe system for identifying released males in the field. Here we describe the creation of strains having both male-specific expression of *bp*^+ ^for pupal sexing, and *PUbnlsEGFP *for identification in field traps.

## Results

**Figure 1:**
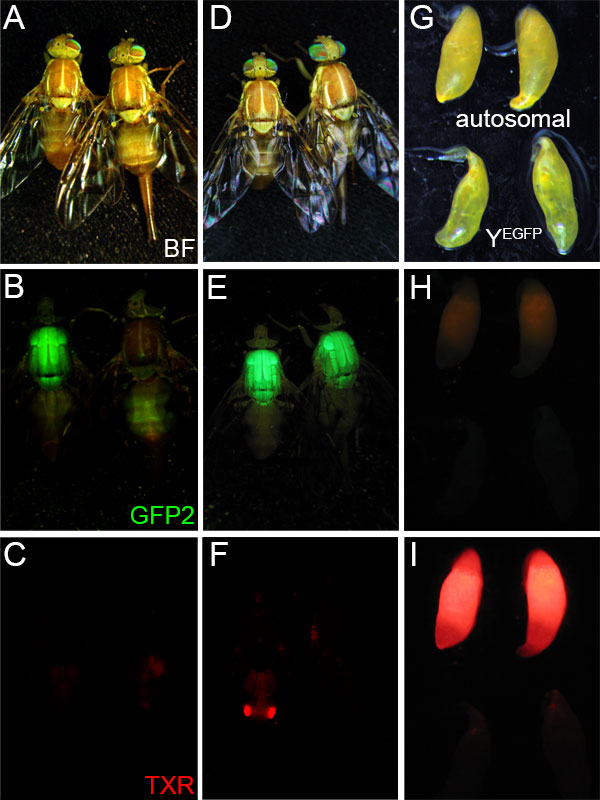
**Y-linked and autosomal fluorescent marker expression in *A. ludens* transformed with pBXL{*PUbnlsEGFP, Asβ2tub-DsRed.T3*}**. The brightfield (BF; A, D, G) and epifluorescent EGFP (GFP2; B, E, H), and DsRed (TXR; C, F, I) phenotypes of: a Y^EGFP ^male (left) and female (right) shown in panels A, B, and C; an autosomal insertion (unmapped) strain male (left) and female (right) shown in panels D, E, and F; and testes from a Y^EGFP ^and autosomal insertion strain male shown in panels G, H, and I. See Methods for details on epifluorescent microscopy and filter sets.

***Y^EGFP ^vector remobilization*. **Segregation analysis of lines transformed with the *piggyBac *transformation vector, pBXL{*PUbnlsEGFP, Asβ2tub-DsRed.T3*} (see Additional file 1) [13], indicated that one line, Y^EGFP^, was Y-linked due to *PUbnlsEGFP *fluorescent marker expression being limited to males, and segregation analysis showing male-specific inheritance (Figure 1). However, the sperm-specific expression of *Asβ2tub-DsRed.T3*, observed in four other autosomal insertion lines, was not observed in the Y-linked line (Fig. 1A-C). The structural integrity of the *Asβ2tub-DsRed.T3 *vector construct in Y^EGFP ^was verified by PCR sequencing (see Additional file 2), indicating that this was not due to a mutation or rearrangement.

Therefore, to determine whether suppression of *Asβ2tub-DsRed.T3 *was due to a chromosomal position effect the vector was re-mobilized by injection of *phsp-pBac *transposase helper plasmid into 832 embryos from the Y^EGFP ^hemizygous line. Of these, 40 G0 surviving males were individually crossed to three wild type females, resulting in three G1 lines where adult males expressed both thoracic EGFP and testis-specific DsRed fluorescence (Fig. 1D-F), whereas the remaining 37 fertile matings expressed only EGFP. Segregation analysis of crosses to wild type indicated that the DsRed fluorescent lines resulted from remobilization into autosomal loci. In addition to PCR transgene sequencing in the Y^EGFP ^line and ME8 autosomal line, derived from the vector remobilization in Y^EGFP ^(see Additional file 2), this verifies the functional integrity of the original Y-linked vector insertion, and suggests that Y chromosome suppression of *Asβ2tub-DsRed.T3 *expression had occurred. Transposon vector remobilizations typically result in local insertions (or 'hops') into sites within the same linkage group (which facilitates transposon mutagenesis strategies) [23]. It is not unlikely that local hops occurred in this remobilization as well, which would not have been recognized if *Asβ2-tubulin *promoter suppression was a general attribute of Y linkage, and not limited to a specific locus (or loci).

**Figure 2:**
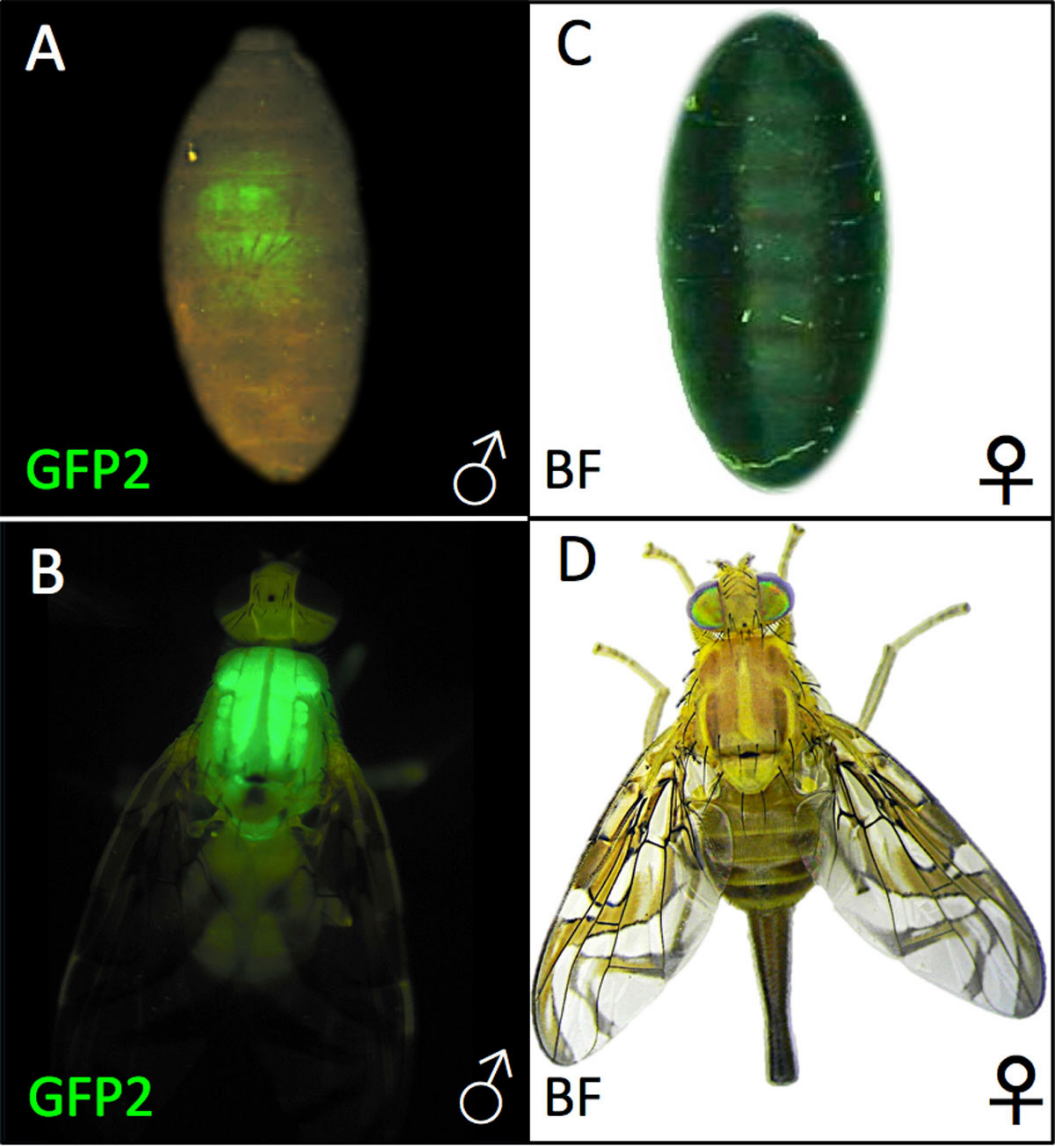
**Phenotypes of T(Y^EGFP^/*bp^+^*) pupal and adult males and females**. *A. ludens *T(Y^EGFP^/*bp^+^*) male *bp^+ ^*brown pupa (A) and male adult (B) under epifluorescent GFP optics, and a female *bp*^- ^black pupa (C) and female adult (D) under brightfield optics. All T(Y^EGFP^/*bp^+^*) males express the wild type brown pupal and EGFP phenotype, while all females express the mutant black pupal phenotype and lack EGFP fluorescence (not shown).

***Translocation Y-EGFP/bp^+ ^strain development*. **To create a *black pupae *sexing strain marked with male-specific *PUbnlsEGFP *expression to identify released males in traps, the Y^EGFP ^strain was used as a host strain for a *bp^+ ^*translocation induced by γ-irradiation as described in Methods. From Y^EGFP ^irradiated pupae, 900 adult males were screened, from which five potential lines were selected where all females had the mutant *black pupae *(*bp^-^*) phenotype, and all males had the brown pupae (*bp^+^*) wild type phenotype, in addition to green fluorescence observed under epifluorescent optics (Table 1; Figure 2).

**Table 1:** F_2 _progeny of *Y^EGFP^/bp^+ ^*translocation males

Lines	Pupae		F_2 _adults*				adult eclosion (%)
				
			*bp^+^*		*bp^-^*		
		
	*bp^+^*	*bp^-^*	male	female	male	female	
T(Y^EGFP^/*bp^+^*)-1	57	46	51	0	0	40	88.35

T(Y^EGFP^/*bp^+^*)-2	34	30	21	0	0	19	62.50

T(Y^EGFP^/*bp^+^*)-3	66	51	62	0	0	45	91.45

T(Y^EGFP^/*bp^+^*)-4	73	57	40	0	0	18	44.62

T(Y^EGFP^/*bp^+^*)-5	39	25	35	0	0	13	75.00

* adults emerging from indicated pupal phenotypes

***Evaluation of the T(Y^EGFP^/bp^+^) strains*. **Life fitness parameters for the five translocation strains were evaluated by observing the survival of 1,000 embryos through life stages from larval hatching to adulthood. Overall survival from the egg stage to adulthood was 17.6% in line T(Y^EGFP^/*bp*^+^)-1 to 36.4% in line T(Y^EGFP^/*bp*^+^)-4, which was comparable to 38.1% survival in the Tapachula-7 control strain already being mass-reared for SIT programs. Line T(Y^EGFP^/*bp*^+^)-3 had a similar survival rate of 33.4% (Table 2).

**Table 2:** Fitness of translocation and reference lines.

Line	egg hatch	egg to larvae survival	larvae to pupae survival	pupae to adult survival	males	egg to adult survival
Wild type	93.00 ± 0.69 a	81.00 ± 2.32 a	95.99 ± 2.03 ab	98.28 ± 0.63 a	0.45 ± 0.01 b	76.60 ± 3.25 a
T(Y^EGFP^/bp+)-1	42.80 ± 4.74 d	19.60 ± 1.60 e	99.23 ± 1.95 a	90.89 ± 2.56 a	0.61 ± 0.03 a	17.60 ± 1.44 d
T(Y^EGFP^/bp+)-2	58.90 ± 4.96 bc	27.10 ± 3.44 de	98.80 ± 0.51 a	90.39 ± 1.71 a	0.54 ± 0.03 ab	24.10 ± 3.12 cd
T(Y^EGFP^/bp+)-3	56.20 ± 2.38 c	35.60 ± 1.71 cd	97.65 ± 0.79 ab	95.95 ± 1.67 a	0.55 ± 0.02 ab	33.40 ± 1.84 bc
T(Y^EGFP^/bp+)-4	94.90 ± 0.65 a	62.60 ± 1.78 b	94.24 ± 1.13 ab	61.84 ± 2.49 b	0.62 ± 0.02 a	36.40 ± 1.66 bc
T(Y^EGFP^/bp+)-5	66.90 ± 1.30 bc	36.70 ± 1.95 cd	89.95 ± 3.56 b	61.27 ± 12.72 b	0.52 ± 0.02 ab	29.80 ± 1.91 bc
T(Y/bp+)-7 "Tapachula-7"	71.80 ± 3.18 b	43.80 ± 2.59 c	89.95 ± 3.56 ab	89.86 ± 1.57 a	0.50 ± 0.02 ab	38.10 ± 2.07 b

***Survival tests***: Egg hatch (F_6,63 _= 39.32, P < 0.0001); egg to larvae (F_6,63 _= 87, P < 0.0001); larvae to pupae (F_6,63 _= 104.21, P = 0.0135); pupae to adult (F_6,63 _= 9.39, P < 0.0001); male proportion (F_6,63 _= 4.61, P = 0.0006); egg to adult (F_6,63 _= 69.38, P < 0.0001)

The integrity of translocation strains can often be compromised by recombination, especially between sequences within the translocated autosome. When this occurs in sequences proximal to the centromere, the mutant and WT alleles can be exchanged resulting in a breakdown of the sexing system. To assess such recombination in the T(Y^EGFP^/*bp^+^*) lines, they were maintained without selection for four generations and then screened for an exchange of the *bp^+^* and *bp^- ^*phenotypes in males and females. In the T(Y^EGFP^/*bp^+^*)-1 and -2 lines recombinant individuals were not detected, while the T(Y^EGFP^/*bp^+^*)-3, -4 and -5 lines exhibited 0.28% (1 male *bp^-^*), 0.23% (1 female *bp^+^*) and 1.74% (4 male *bp^-^*; 2 female *bp^+^*) recombinant frequencies, respectively. These frequencies are considerably higher than the 0.05% frequency for Tapachula-7 [21], and is most likely a function of the distance between the *bp *allele and translocation breakpoint, which is expected to increase with distance [24]. Since the strains exhibiting recombinants were also the most highly viable, induction of an inversion in this region to suppress recombination, as has been achieved for the medfly VIENNA-8 translocation sexing strain [24], may be considered. Selection of additional translocation lines having strong viability and minimal recombination is also feasible.

## Discussion

Here we report the creation of an *A. ludens *transgenic line with a *piggyBac *transformation vector that includes fluorescent protein markers useful for identifying released males in the field and wild females that have mated with the released males. Notably, the vector insertion site is Y-linked, so that a sexing line could be created by translocating the wild type allele for the *bp *mutation onto the Y chromosome, allowing the separation of black pupal (*bp^-^*) females from brown pupal (*bp^+^*) males during rearing.

Use of pupal color markers in Y-translocation strains has been an efficient means of creating sexing strains in tephritid flies [18,19]. Recessive mutations resulting in pupal phenotypes exhibiting darker or lighter coloration than wild type are relatively common, and translocations of their wild type allele to the male-specific Y chromosome are straightforward to create and select. Relatively inexpensive rice sorters can then be used to efficiently separate large numbers of wild type male pupae from mutant females. One drawback is that, typically, pupal markers do not confer an adult phenotype (or one that is easily identifiable), so that identification of released males depends upon the use of fluorescent powders that can be unreliable (due to loss from grooming or transfer to wild males), and a health risk to workers involved in rearing and release [22]. Thus, the male-specific Y-linked fluorescent protein transgene marker provides a reliable means of identifying released male adults in traps, a secondary means of verifying pupal sex if cuticle coloration is ambiguous, and a rapid means of identifying putative recombinants (having an EGFP/*bp^- ^*phenotype). If Y-linked fluorescence is detectable in embryos or early stage larvae, it may be eventually useful as a means to select males by automated fluorescence-based sorters early in development [9], thereby eliminating females previous to rearing to the pupal stage, which is costly and inefficient.

The Y-linked transformant line was originally selected during a previous transformation experiment, where both the *polyubiquitin*-regulated EGFP body color marker and the *β2-tubulin*-regulated sperm marker were easily identifiable and distinguishable in autosomal integrations [13]. However, while the Y-linked *PUbnlsEGFP *marker was strongly expressed and reliably detected in males, the *Asβ2tub-DsRed *marker was not visibly detectable, which we presume is the result of suppressed transcription since its sequence integrity has been verified. This is unfortunate since it eliminates the ability to identify females that have mated with the transgenic males by identifying fluorescent sperm stored in their spermathecae. However, remobilization of the Y-linked integration to autosomal sites restored *Asβ2tub-DsRed *expression, which may be similarly achieved in T(Y^EGFP^/*bp*^+^) strains by a local remobilization of the pBXL{*PUbnlsEGFP, Asβ2tub-DsRed.T3*} vector to the translocated autosome, thereby maintaining male-specificity. Alternatively, an autosome carrying the vector transgene could be crossed into the translocation line, thus providing both fluorescent markers.

Beyond an unusual phenomenon, the Y-specific suppression of the *Asβ2tubulin *promoter may, nevertheless, have important implications for how the male germ-line is regulated by the Y chromosome in tephritids. Position effect variegation (PEV), resulting from suppression of gene expression typically affecting euchromatic genes positioned proximal to or within heterochromatin, is well documented [25]. Differential promoter regulation by PEV is less well established, but evidence exists in *D. melanogaster *for the Y chromosome having a general suppressive effect on PEV [26-28], and for specific regions of the Y chromosome having a positive trans-activator function specifically for transcription of male germ-line genes [29]. If this type of activity occurs in mexfly, it is conceivable that the transgene vector integration may have disrupted Y-activation of the *Asβ2-tubulin *promoter, but if so, other germ-line genes (including the native *A. ludens β2-tubulin *gene) also should have been affected resulting in diminished fertility, which was not apparent. Remobilization of the transgene could have also resulted in local hops within the Y, with the expectation that a site or region-specific position effect on the original insertion would be less effective in some remobilized Y-linked lines, which was also not apparent. Thus far, the specific suppression of a Y-linked *β2-tubulin *gene promoter, or any other promoter, is a unique observation. It will be important to determine whether this is the result of a gene expression regulatory function that is specific to a particular Y-linked locus or region, or a chromosome-wide effect for the chromosome, and whether other male germ-line specific genes are similarly affected.

## Methods

***Insect strains*. **The *black pupae *(*bp^-^*) mutant strain was originally isolated from *A. ludens *flies mass-reared at the MOSCAFRUT facility. The pBXL{*PUbnlsEGFP, Asβ2tub-DsRed.T3*} transgenic strains were created as previously described [13], with the Y^EGFP ^strain having a Y-linked integration based on segregation analysis. Transgenic flies were screened by epifluorescence microscopy for DsRed (TXR filter: ex: 560/40; em: 610 LP) and EGFP (GFP2 filter; ex: 480/40, em: 510 LP) fluorescence. The wild type Chiapas strain was originally collected from infested fruit in the state of Chiapas, Mexico, and the genetic sexing strain "Tapachula-7" was created as described [21].

***Plasmids*. **The pBXL{*PUbnlsEGFP, Asβ2tub-DsRed.T3*} *piggyBac *transformation vector (plasmid #389) used to create the Y^EGFP ^strain was described previously (see Additional file 1) [10,13]. The *piggyBac *transposase helper plasmid, *phsp-pBac*, used to remobilize pBXL{*PUbnlsEGFP, Asβ2tub-DsRed.T3*} in Y^EGFP^, was described previously [30].

**pBXL{PUbnlsEGFP, Asβ2-tub-DsRed.T3} *remobilization*. **Remobilization of the pBXL{*PUbnlsEGFP, Asβ2tub-DsRed.T3*} vector in Y^EGFP ^followed typical germ-line transformation procedures for *Anastrepha *species [3,13], except that Y^EGFP ^G0 embryos were injected solely with 500 µg/ml of *phsp-pBac *helper plasmid. Eclosed G0 adults were backcrossed in small groups to Chiapas wild type host flies, with resulting G1 adult progeny examined under epifluorescence optics for EGFP and DsRed expression. Autosomal or sex-linkage of vector insertions were determined by outcrossing G2 and G3 males and females to wild type. Chromosomal insertions of pBXL{*PUbnlsEGFP, Asβ2tub-DsRed.T3*} determined to be Y-linked due to male-specific marker expression were designated as Y^EGFP^.

***Y^EGFP ^/bp^+ ^translocation strain*. **Pupae from the Y^EGFP^; *bp^+^/bp^+ ^*strain were γ-irradiated with 30 Gy using Cobalt^60^, with newly eclosed males crossed to homozygous *bp*^-^/*bp*^-^mutant females. Phenotypic wild type (brown) F_1 _males, having the genotypes Y^EGFP^; *bp^+^/bp^- ^*or T(Y^EGFP^, *bp^+^*); Df(*bp^+^*)/*bp^-^*, were backcrossed to *bp^-^*/*bp^- ^*females in single pair matings, with F_2 _T(Y^EGFP^, *bp^+^*) translocation lines identified by those having all males eclosing from brown pupae (*bp*^+^) (expected in all lines), but where all females eclosed from black pupae (*bp*^-^), versus black and brown female pupae generated from non-translocation males. F_2 _females inheriting the Df(*bp^+^*) autosome from translocation males were lethal due to aneuploidy, and thus only *bp*^-^/*bp*^- ^females survived. Male-specific expression of *PUbnlsEGFP *also indicated that the pBXL{*PUbnlsEGFP, Asβ2tub-DsRed.T3*} Y-linked insertion was not deleted by the translocation, and these lines were designated as T(Y^EGFP^/*bp^+^*).

***Life fitness test*. **All translocation lines were inbred with approximately 1,000 eggs from each line put on artificial diet in groups of 100 eggs, with larvae and pupae collected and recorded [31]. Pupae were sexed by pupal color that was verified after adult eclosion. The same procedure was applied as a control to the Chiapas wild type and Tapachula-7 strains. Statistical analysis was carried out comparing the Y^EGFP ^translocation strains, the Tapachula-7 strain and the wild type *A. ludens *strain by analysis of variance (ANOVA) and Tukey-Kramer tests [32].

***PCR analysis***. To verify the integrity of the *Asβ2tub-DsRed.T3 *marker transgene in autosomal and Y-linked vector integrations, genomic DNA from the autosomal ME8 and Y-linked T(Y^EGFP^, *bp^+^*) lines was isolated for PCR reactions using the primer pair P15 (GGTGGAGCTCCAGCTTTTGTTCC) / MFS-10 (ACGACCGCGTGAGTCAAAATGACG) and Platinum Taq polymerase (Invitrogen). PCR was performed on both genomic samples and the control AH389 vector plasmid using the following conditions: 1 min at 95°C; 5 cycles of 15 s at 94°C, 20 s at 65°C (-2°C/cycle), 2.5 min at 72°C; 30 cycles of 30 s at 94°C, 45 s at 56°C, 2.5 min at 72°C; and 3 min at 72°C. All 2.4 kb fragments were subcloned in pCR4 vector (Invitrogen) and sequenced at Macrogen using the oligos M13F, M13R and P17 (CCGTCGGAGGGGAAGTTCACG). Multiple sequence alignments were performed in Geneious 7.1 (Biomatters, Ltd.) using the standard Geneious Alignment algorithm.

## Competing interests

The authors declare that they have no competing interests.

## Authors' contributions

JSM, AMH, MFS, and CSZ-C conceived of the study, its design and coordination, and JSM carried out the transformation and vector remobilization studies and creation of the translocation strain. AMH created the vector and helper plasmids and MFS carried out PCR analysis. AMH, MFS and JSM wrote the manuscript, for which the final draft was read and approved by all authors.

## Supplementary Material

Additional file 1 Schematic (to scale) of the pBXL{*PUbnlsEGFP, Asβ2tub-DsRed.T3*} transformation vector. Click here for file

Additional file 2 Integrity of *Asβ2tub-DsRed.T3 *marker transgene. A multiple sequence alignment of PCR sequenced transgene vector fragments from genomic DNA from the Y-linked Y^EGFP ^and autosomal ME8 transformant lines, and the pBXL{*PUbnlsEGFP, Asβ2tub-DsRed.T3*} plasmid vector. This verifies the integrity of the marker transgene in the two transformant lines based on 100% identity among the sequences. Click here for file
