# Supplementary figures and images for: Male-specific Y-linked transgene markers to enhance biologically-based control of the Mexican fruit fly, Anastrepha ludens (Diptera: Tephritidae)
Source: BMC Genet. 2014 Dec 1;15(Suppl 2):S4. doi: 10.1186/1471-2156-15-S2-S4 (PMC4255795; doi:10.1186/1471-2156-15-S2-S4)

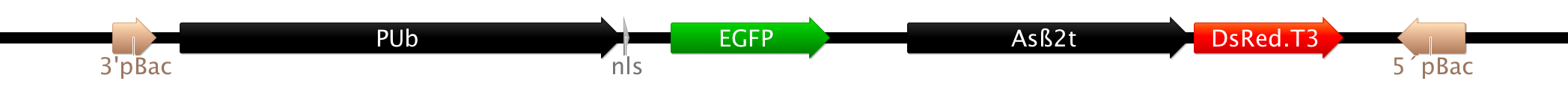

Supplement: Additional file 1 — Schematic (to scale) of the pBXL{PUbnlsEGFP, Asβ2tub-DsRed.T3} transformation vector. [file 1471-2156-15-S2-S4-S1.tif]

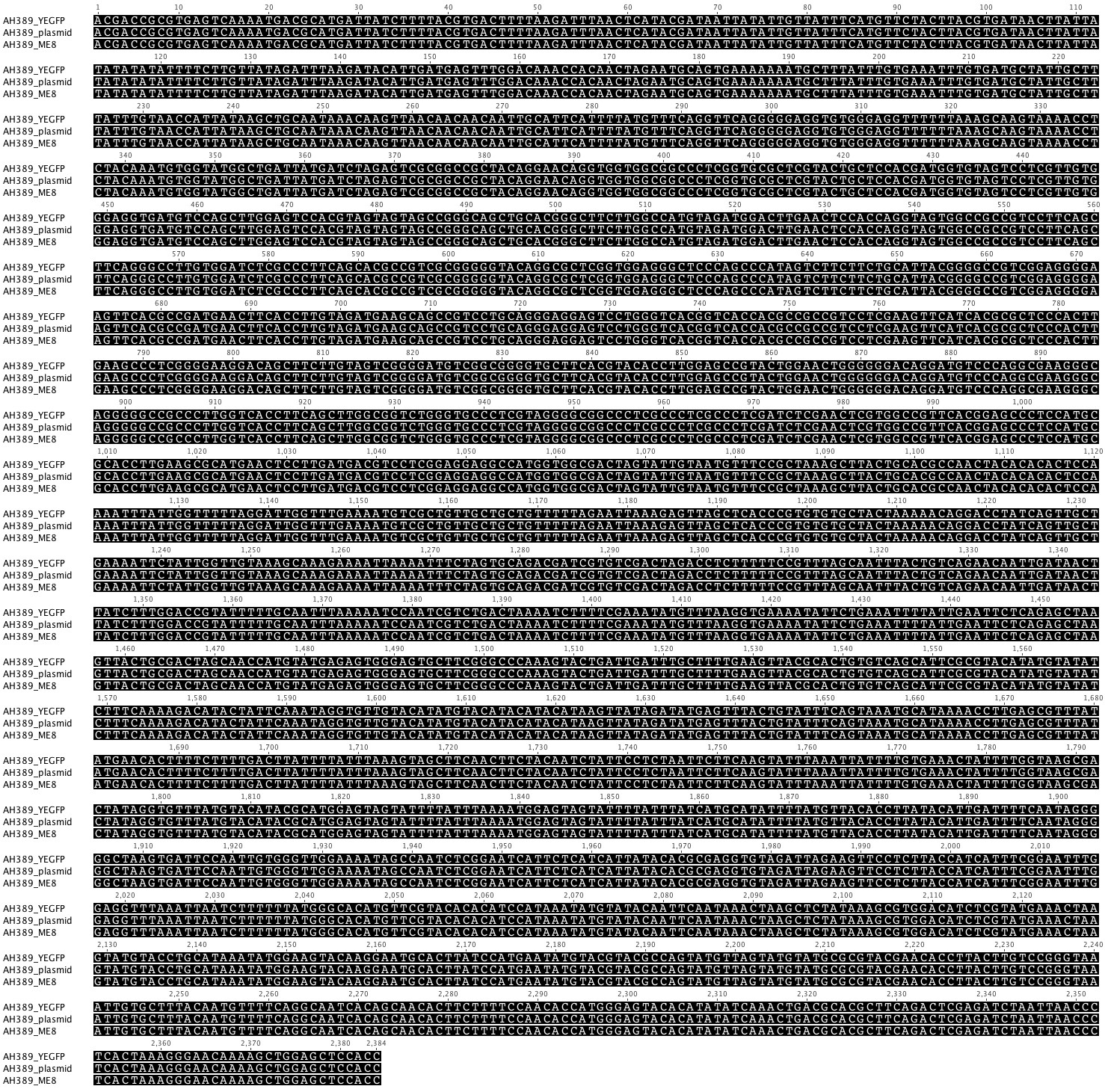

Supplement: Additional file 2 — Integrity of Asβ2tub-DsRed.T3 marker transgene. A multiple sequence alignment of PCR sequenced transgene vector fragments from genomic DNA from the Y-linked YEGFP and autosomal ME8 transformant lines, and the pBXL{PUbnlsEGFP, Asβ2tub-DsRed.T3} plasmid vector. This verifies the integrity of the marker transgene in the two transformant lines based on 100% identity among the sequences. [file 1471-2156-15-S2-S4-S2.tif]
